# How does internet use affect the sense of gain in older adults? A moderated mediation model

**DOI:** 10.3389/fpsyg.2025.1538086

**Published:** 2025-02-26

**Authors:** Yi Yang, Ying Ni

**Affiliations:** School of Public Administration, Xi'an University of Architecture and Technology, Xi’an, China

**Keywords:** sense of gain, Internet use, the elderly, mediating effects, moderating effect

## Abstract

In the context of the digital era, Internet use is of major significance for enhancing the sense of gain among elderly people and enabling them to share in the development dividend of the digital society. Based on CGSS 2021 (*N* = 2,929), this study explores the impact of Internet use on the sense of gain in the elderly, and a moderated mediation model was constructed to explore the mediating role of perceived social justice and the potential moderating role of social status. The results showed that (1) Internet use in general significantly enhanced elderly’s sense of gain. Among this group, economic sense of gain (ESG) was significantly enhanced, political sense of gain (PSG) was positively affected but not significantly, while security sense of gain (SSG) was negatively affected. (2) A sense of social equity plays a mediating role in the impact of Internet use on the sense of gain among older adults. (3) Social status does not play a moderating role in the latter half of the path of the mediation model. Meanwhile, heterogeneity analysis revealed that Internet use positively and significantly affects the sense of gain among elderly people with low household incomes, high levels of literacy, an urban household registration, and a spouse. The research conclusion of this article provides strong empirical support for solving the problem of digital integration among the elderly and effectively enhancing their sense of achievement.

## 1 Introduction

Population aging is an important global economic and social trend (Mason et al., 2022; Maestas et al., 2023). Data from China’s National Bureau of Statistics indicated that by the end of 2023, China’s elderly population (those aged 60 and above) exceeded 296 million, accounting for 21.1% of the total population (National Bureau of Statistics of China, 2025). This means China is facing a serious issue of population aging (Bao et al., 2022; Ren et al., 2023). As people age, they face a series of challenges such as high incidence of various diseases, limited mobility, and social isolation, which profoundly affect their physical and mental health status (Rony et al., 2024). Meanwhile, the rapid development of digital technology means the Internet is increasingly penetrating the lives of the elderly. According to the 55th Statistical Report on the Development of Internet in China, the Internet penetration rate among the elderly in China was expected to reach 52.5% by December 2024 [China Internet Network Information Center (CNNIC), 2025]. Given the dual background of the convergence of digitalization and aging, access to and the use of Internet technology provide entertainment and hobbies for the elderly (Llorente-Barroso et al., 2021); help them keep in touch with family members; and enable them to actively use medical resources (Sen et al., 2022). These aspects significantly affect the health and wellbeing of this population segment (Lee, 2024). Therefore, key measures to promote the realization of the goals of “healthy aging” and “active aging” are, firstly, helping older persons to use the Internet more conveniently and safely, and, secondly, recognizing and responding to the practical needs of this group at both the material and spiritual levels (Stelcer et al., 2023; Bernardo et al., 2022).

Given the realities brought about by the development of an aging population, China has made the “sense of gain” an important evaluation criterion for measuring economic and social development and benefits for citizens (Duan and Li, 2022; Yue et al., 2024). Since 2015, when General Secretary Xi Jinping proposed the concept, the “sense of gain” has not only been frequently mentioned in Chinese government documents but has also become an important basis for China’s proactive response to its aging society (Puqu and Chengyuan, 2018). Traditional studies often use established concepts such as “happiness” and “subjective quality of life” to evaluate quality of life (Santos et al., 2007; Prati, 2022). However, a positive response to the problem of aging must not focus on only one aspect of the material or spiritual wellbeing of the elderly; a comprehensive grasp is required of the actual needs of this group. The sense of gain refers to the subjective feeling of “gain,” which is based on “objective gain.” As a novel analytical concept, “sense of gain” combines the objective gains and subjective feelings of social groups, and it is an important analytical tool for study of economic and social development and national governance. Thus, in this context of aging, “sense of gain” provides a new explanatory path for exploring the “digital existence” of the elderly, as attempted in this study. Therefore, “sense of gain” is an innovative concept that integrates subjective feelings and objective gains, warranting further exploration of the effects of Internet use on the elderly’s sense of gain.

With the continuous development of digital technology, Internet technology is considered one of the most important programs to actively address the issue of an aging society (Toros et al., 2024). However, the rapid diffusion of Internet technology superimposed on the global trend of population aging has led to widening welfare gaps among older groups in the digital age (Larasati et al., 2023; Repetti and Fellay-Favre, 2024). A review of the literature revealed several theoretical debates concerning how Internet use affects the wellbeing of older adults, resulting in two main conclusions: “optimistic promotion” and “negative inhibition.” On the one hand, scholars have pointed out that digital technologies can help empower the public and facilitate older persons’ access to information services and participation in social life (Thangavel et al., 2024). Internet use has a positive impact on the subjective wellbeing of older persons, and it can improve their social adjustment (Lv et al., 2020; Tirado-Morueta et al., 2020; Yu et al., 2024; Jiajun et al.,2022). On the other hand, some scholars have emphasized that senior citizens are less capable of accepting Internet life (Schehl et al., 2019) and that issues like the digital divide, fear, and privacy risks tend to harm the lives of older people (Wu et al., 2015).

To summarize, the impact of Internet technology on the sense of acquisition among the elderly is both complex and uncertain. In the context of the digital era, exploring the intrinsic connection between Internet use and the sense of acquisition among the elderly is linked to the quality of life of individuals in this group. It also represents an important active response to the challenges of population aging and promotes the harmonious development of society. The literature outlined above establishes a solid foundation for the current research. However, certain issues have not been considered and warrant further exploration. First, most existing studies focus on a single dimension, such as the material satisfaction or spiritual pleasure of the elderly. Moreover, researchers have often deployed a traditional indicator (happiness) and focused on assessing the degree of psychological satisfaction among the elderly. This has produced a relatively limited research perspective that makes it difficult to comprehensively reveal the deeper relationship between the elderly’s Internet use and overall wellbeing. Secondly, as an innovative concept combining subjective feelings and objective acquisition, the term “sense of gain” has rich and multidimensional connotations that encompass both direct perceptions of quality of life among older persons and comprehensive evaluations of social progress. However, this dynamic perspective is often overlooked in the literature. Therefore, data from the China General Social Survey 2021 (CGSS2021) were used in this study to empirically examine the relationship between Internet use and the elderly’s sense of gain. Based on this, the authors analyzed the mechanisms by which perceptions of social fairness and social status influence this relationship. The study also aimed to assess differential manifestations of these effects among older adults who have different characteristics.

Compared to the existing studies, this new paper makes several marginal contributions. First, it explores the comprehensive impact of Internet use on the elderly’s sense of gain, which has important theoretical significance for research on elderly wellbeing in the context of the digital age. Second, utilizing the CGSS2021 survey data produced major practical advances in measuring three aspects of the sense of access among the elderly from a micro-individual perspective: economic access, political access, and security access. The survey data were also used to examine, with regard to Internet use, four separate aspects of heterogeneity―family income, education level, urban and rural hukou, and marital status―that influence the sense of gain among the elderly. The purpose was to provide empirical support that would enable an enhanced sense of gain among this group.

The remainder of this study is arranged as follows: Section 2 discusses the relevant literature on the impact of Internet use on the sense of gain among the elderly. The section also presents the research hypotheses developed on this basis. Section 3 includes the research design, including the data sources, variable selection, and model setting. Section 4 reports the results of the empirical analysis. Section 5 contains the discussion, while Section 6 forms the conclusion.

## 2 Literature review and research hypotheses

### 2.1 The impact of internet use on the elderly’s sense of gain

The sense of gain is a composite concept with a strong local flavor. A sense of gain differs from a sense of wellbeing or satisfaction in that it stems from the individual’s fulfillment of their physical and spiritual needs (Tan et al., 2020; Zhou et al., 2021). According to Marx, the satisfaction of human needs is a “system of needs” that integrates multiple aspects and levels of needs (Heller, 2018). Considering the main paths to satisfying individual needs, the concept of “rationality” proposed by Max Weber distinguishes between value rationality and the instrumental rationality of the subject of action (Cockerham et al., 1993). According to this autonomous agency for individual needs, the satisfaction of needs is not a completely independent individual process, but it needs to be achieved in the appropriate social context. The need for self-actualization will eventually be further stimulated by the satisfaction of basic needs (Compton, 2024). Understanding “sense of gain” from different levels of demand satisfaction, sense of gain refers to the subjective evaluation made by multi stakeholder subjects in the process of economic and social development, based on the satisfaction of their own needs and actual gains (Yabin, 2024). More specifically, economic development, political participation, and the realization of safety and security are important foundations in sourcing a sense of gain (Yang and Zhang, 2019). Therefore, the sense of gain among the elderly can also be realized and measured through the economic sense of gain (ESG), political sense of gain (PSG), and security sense of gain (SSG).

Scholars have found that the use of Internet technology significantly impacts the elderly’s sense of gain, and policy recommendations have been proposed to enhance this sense of gain effectively. However, analyzing the relationship between Internet use and the elderly’s sense of gain from different perspectives has led to two competing logical views of the impact of Internet use on the sense of gain in this group: strengthening and weakening. In the following section, the two perspectives are arranged separately and different research hypotheses are proposed.

Compared with traditional ways of accessing information, the Internet has more quickly reshaped the past living habits and inherent habits of the elderly. Increasing numbers of elderly people use the Internet for social activities, online shopping, and entertainment consumption (Wilson-Nash and Pavlopoulou, 2024). Starting from the social support theory, some scholars believe that the Internet has broadened the elderly’s scope of socialization, potentially enabling the subjective integration of the elderly into society and reducing their sense of loneliness and negative emotions (Habibi et al., 2021; Zheng and Zhang, 2023). Other scholars have argued that older people who actively participate in online life are less disconnected from modern society and that using the Internet enhances older people’s levels of social adjustment (Liu et al., 2021). Owing to the inclusive effect of the Internet, network technology can help older persons improve their knowledge and accumulate social capital, effectively reducing the cost of re-employment activities for this group (Hinrichs, 2021). In addition, a longitudinal cohort study of older people in the United Kingdom found that the Internet provides the level of information and skills that older people need to maintain health management, which is important for improving their physical and mental health (Heponiemi et al., 2022). In short, the majority of scholars reporting optimism about Internet use affirm its contribution to older adults’ sense of gain. Internet technology has unique advantages in terms of interactivity, sharing, and the efficiency of information dissemination, which technically enhance the capability of older persons to share in the development of modern society; moreover, the needs of older persons are met in terms of physical and mental health, social integration, and other aspects. Accordingly, the following hypothesis was formulated:

*H1a*: Internet use positively contributes to the elderly’s sense of gain.

However, some studies suggest that Internet use negatively affects older people’s sense of gain through “substitution effects” and “adverse use effects.” More specifically, The former indicates that Internet use reduces face-to-face interaction and communication among older adults, increases the sense of disconnection between interpersonal interactions, and hinders the individual expression of emotions and the maintenance of real relationships (Sen et al., 2022). Meanwhile, “adverse use effects” reflect the possibility of over-reliance on Internet information, which can have a crowding-out effect on the real-world participation of older persons (Benvenuti et al., 2020). Furthermore, when older adults lack self-control and are addicted to using the Internet, their sense of access may be significantly negatively impacted. Examples include addiction to questionable short-form video content or mobile video games (Shen et al., 2023). Elderly people often cannot independently distinguish between true and false information, and they are prone to becoming both victims and disseminators of online rumor and false information (Tsfati et al., 2020). These issues will greatly weaken the sense of gain among this group. In addition, differences in individual digital literacy have led to the creation of a digital divide, resulting in some older people experiencing greater difficulty in accessing public social services and an apparent sense of relative deprivation (Zhang et al., 2024). For example, when older people need to access public services through online self-service, the overly complex digital skills requirements often create an increased administrative burden (Madsen et al., 2022). In summary, Internet use has a similarly adverse effect on older adults’ sense of gain. Based on this discussion, the following hypothesis is examined in this paper.

*H1b*: Internet use has a negative inhibitory effect on the elderly’s sense of gain.

### 2.2 The mediating role of a sense of social equity

A sense of social equity means an individual’s basic perception or value judgment about the state of social equity (Hvidberg et al., 2023). The level of this perception not only depends on the individual’s reflection of the real social environment but also is influenced by personal values and social culture. Thus, an individual’s perception of equity interacts with the group when confronted with particular social scenarios as well as the status quo. Studies have provided an exhaustive typology of social equity perceptions, examples of which include institutional equity, economic equity, security equity, and political equity (Li, 2019). Internet use, it has been argued, can lead to more discussion of real matters and a greater understanding of the political community, thus promoting a sense of social equity (Mehra, 2023). Internet participation allows older people to better understand social policies and helps to enhance their perception of social equity. Moreover, older people, as opposed to younger people, respond positively to social inequalities as they age (Weiss et al., 2022). Among older adults, Internet use can more effectively enhance perceptions of social equity. Therefore, the following hypothesis was proposed:

*H2*: Internet use positively contributes to older adults’ sense of social equity.

A review of the literature shows that social equity can affect the sense of gain. The elderly perceive social equity more in terms of infrastructure friendliness, social equity, and old age security. Information processing theory emphasizes that an individual’s sense of social equity, like a citizen’s subjective perception of the social environment, can affect their emotional antecedent evaluations and emotional responses (Tybout et al., 1981; Wang et al., 2023). Even when confronted with the same information, people tend to select social information that is consistent with their perceptions of processing. Therefore, if older adults have a poor social equity perception status, they may be skeptical of positive information on the Internet and strongly identify with negative information, ultimately leading to a decline in the sense of gain. Numerous empirical studies have also shown that if the sense of social equity is reduced, then the public’s sense of gain, happiness, and sense of security will be greatly reduced as well (Zheng and Zhang, 2023). A sense of social equity is an important expression of and response to older people’s sense of gain. Measures such as the establishment of a sound social distribution system and social security mechanisms can significantly improve the sense of social equity in older persons and contribute to their sense of gain. Therefore, the following hypothesis was proposed.

*H3*: A sense of social equity mediates the impact of Internet use on the elderly’s sense of gain.

### 2.3 The moderating role of social status

Social class has been identified as the most important factor in measuring social equity (Kim et al., 2022). Reasonable social class mobility is an intrinsic requirement of social equity, and the value demands it embodies are the very essence of modern democracies. Generally, groups with higher social status can enjoy good and fair treatment within society, and they even receive an excess supply of material resources. In this way, they gain access to a relative monopoly of social resources. Conversely, groups with low social status may have difficulty accessing basic social equity and often experience deprivation (Iyer and Achia, 2021). Social status is a significant contributor to a sense of social equity, and social status also affects the sense of gain, with lower-middle-class people having the lowest sense of gain (Darity, 2022). Because of this, with full consideration of the current context of social development, and to ensure the scientific nature and generalizability of the research conclusions, social status was included as an influential factor in the model. Therefore, the following hypothesis was proposed.

*H4*: Social status plays a moderating role in the second half of the pathway by which Internet use affects the elderly’s sense of gain through a sense of social equity.

**Figure 1:**
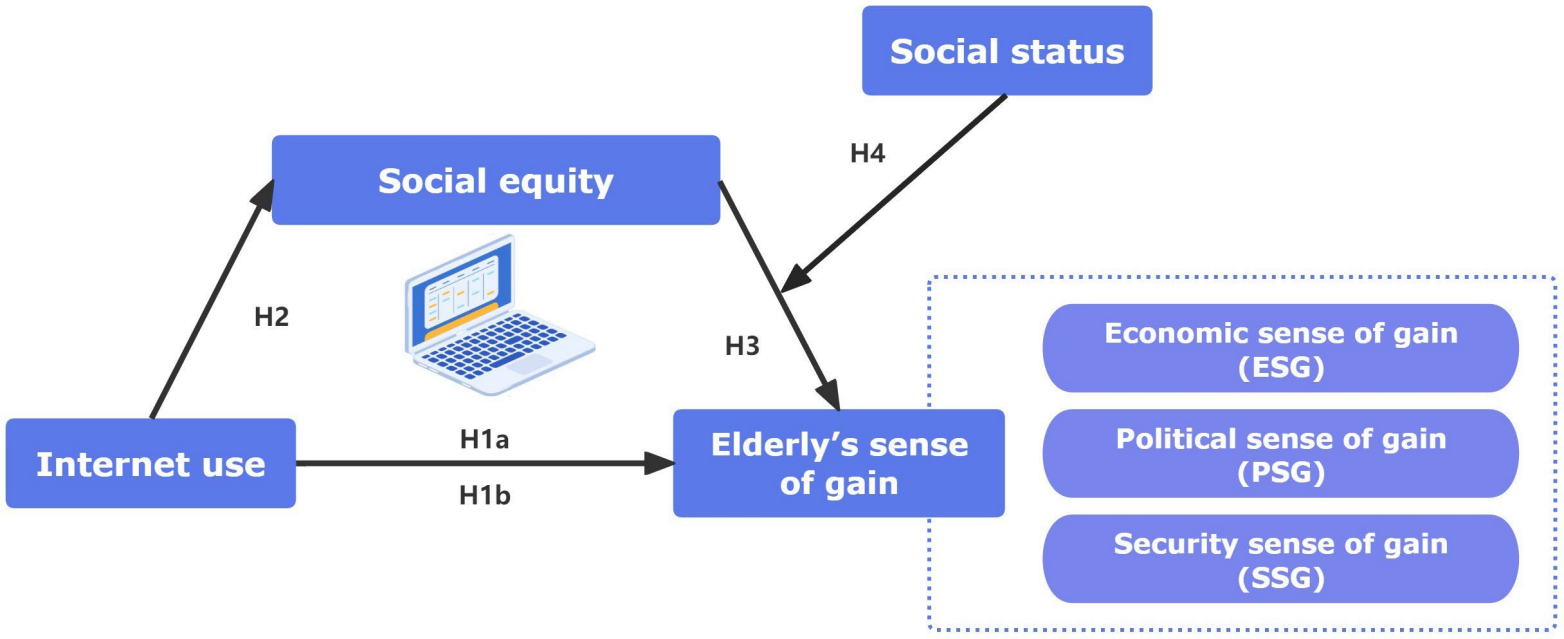
Research hypothesis model.

In summary, a moderated mediation effect model was constructed to examine the mediating (social equity) and moderating (social status) mechanisms of Internet use on the elderly’s sense of gain (see Figure 1).

## 3 Research design

### 3.1 Data sources

The data used in this study came from the 2021 Chinese General Social Survey (CGSS), which was conducted by the China Survey and Data Center of Renmin University, China. The survey uses a continuous cross-sectional questionnaire to describe and analyze Chinese society comprehensively and systematically, revealing changes and development trends in various aspects, including economic, political, social, and cultural. The CGSS2021 database contains 8,148 valid samples nationwide, consisting of 700 variables. Following the definition of age stated in China’s Law on the Protection of the Rights and Interests of the Elderly, elderly people aged 60 and above were selected as the research object. After screening the variables and eliminating the missing values and outliers of the samples, 2,929 valid samples were obtained.

### 3.2 Selection of model variables

#### 3.2.1 Independent variable

Internet use was used as the independent variable. Question A28 in the questionnaire, “In the past year, what was your use of the Internet (including cell phone access)?,” was selected to measure Internet use among the elderly. According to the respondents’ answers, “never” was operationalized as “no” and assigned a value of 0, and “rarely, sometimes, often, or very often” was operationalized as “yes” and assigned a value of 0. “Yes” was assigned a value of 1, as shown in Table 1.

**Table 1:** Sources of indicators.

Target layer	Measure	Sources
Internet use	A28: “In the past year, what was your use of the Internet (including cell phone access)?”	Li et al. (2024) and Zhang et al. (2021)

#### 3.2.2 Dependent variable

The elderly’s sense of gain was chosen as the dependent variable. Drawing on the findings of Yang and Zhang (2019), the sense of gain was further refined into three dimensions: economic sense of gain (ESG), political sense of gain (PSG), and security sense of gain (SSG). For ESG, the questionnaire questions A43e_05: “On balance, in the current society, how would you describe your socio-economic status?” and D35_08: “I am satisfied with my family’s income” were selected and measured. To facilitate the analysis of the model results, the original options of A43e_05 were inverted and assigned the following values: lower = 1, lower-middle = 2, upper-middle = 3, middle = 4, upper = 5. Secondly, for PSG, question E50, “Do you agree that older people have too much political influence?” was selected. The original options were inverted and assigned the following values: completely disagree = 1, disagree = 2, agree = 3, completely agree = 4. Finally, the SSG dimension was developed with reference to Xia et al. (2024), Leung and Wolfson (2021), and Gajda and Jeżewska-Zychowicz (2021). Food safety is directly related to the physical health and general safety of the elderly, so food safety issues may lead to negative emotions such as anxiety and fear. These emotions can reduce the security sense of gain among this group. In contrast, when food safety is guaranteed and highly trusted by the elderly, their psychological sense of security will be enhanced. This helps increase their security sense of gain. Therefore, for this study, question H2_16, “How would you describe the food safety situation in your residential area?,” was selected. The original options were operationalized as follows: very serious = 1, fairly serious = 2, average = 3, not too serious = 4, not serious = 5, and no such problem. This is shown in Table 2.

**Table 2:** Sources of indicators.

Target layer	Criterion layer	Measure	Sources
Elderly’s sense of gain	Economic sense of gain (ESG)	A43e_05: “On balance, in the current society, where does your socio-economic status belong?”	Cui et al. (2023), Barbosa Neves et al. (2018), and Roquet et al. (2023)
		D35_08: “I am satisfied with my family’s income.”	
	Political sense of gain (PSG)	E50: “Do you agree that older people have too much political influence?”	Schehl et al. (2019)
	Security sense of gain (SSG)	H2_16: “Food safety where I live”	Hunsaker and Hargittai (2018)

#### 3.2.3 Mediator

Social equity was used as the mediating variable, based on a single question, A35: “In general, do you think that today’s society is fair?” To explore the elderly’s sense of gain, the options were reassigned as follows: totally unfair = 1, relatively unfair = 2, between fairness and unfairness = 3, relatively fair = 4, completely fair = 5.

#### 3.2.4 Moderator

The moderating variable for this study was social status. Question A43: “In general, in the current society, what level of society are you in?” was selected for measurement. The question was measured using a self-rating scale of 1–10, whereby the higher the value, the higher the individual’s perceived social status.

#### 3.2.5 Control variables

Concerning previous empirical studies and based on the core issues of concern in this study, the control variables selected here included the individual level and the household level. The former covers aspects like age, gender, marital status, census register, education level, and political affiliation; the latter includes annual household income and household size. Annual household income was measured using question A62: “What was the total annual household income of your family in 2020.” For the convenience of analysis, the original unit “yuan” was operationalized as “10,000 yuan.”

### 3.3 Methods

#### 3.3.1 Basic regression model

To test whether Internet use affects the elderly’s sense of gain, an OLS model was constructed for empirical testing:


$$Gain=\beta_{m}+\beta_{n}Internet+\beta_{r}control_{i}+\varepsilon_{i}$$

Where, *Gain* represents the elderly’s sense of gain and *Internet* represents Internet use, *Control_i_* is a set of control variables, and $\varepsilon_{i}$ is the random error term of the regression model.

#### 3.3.2 Mediation effects model

In this paper, the analytical method of stepwise test regression was used to test the model, with social equity as the mediating variable. Stepwise regression analysis, one of the most commonly used tests of mediation, was proposed by Baron and Kenny (1986). Drawing on the research results of Wen and Ye (2014), the benchmark model of this paper was set as follows:


(1) $$Gain=\alpha +\beta_{1}Internet+\overset{8}{\underset{i=1}{\sum }}N_{i}CV_{i}+\varepsilon$$

(2) $$SE=\alpha +\beta_{2}Internet+\overset{8}{\underset{i=1}{\sum }}N_{i}CV_{i}+\varepsilon$$

(3) $$Gain=\alpha +\beta_{3}Internet+\beta_{4}SE+\overset{8}{\underset{i=1}{\sum }}N_{i}CV_{i}+\varepsilon$$

(4) $$Gain=\alpha +\beta_{5}Internet+\beta_{6}SE+\beta_{7}SS+\beta_{8}SE\cdot SS+\overset{8}{\underset{i=1}{\sum }}N_{i}CV_{i}+\varepsilon$$

Where SE denotes social equity, SS denotes social status, *ε* denotes the random error term, SE·SS denotes the interaction term between social equity and social status, and CV_i_ denotes the eight control variables. β1–β8 and Ni denote the effect coefficients of each variable. Equations 1–3 denote tests for mediated effects, and Equation 4 denotes tests for mediation with moderation.

## 4 Results

### 4.1 Descriptive statistics

The statistical results regarding the impact of the elderly’s internet use on their sense of gain are shown in Table 3. The following statistical findings were obtained: (1) Concerning the independent variables, the mean value of Internet participation among the elderly is 0.7730, indicating that 77.3% of this group are involved in Internet use. (2) In terms of the dependent variables, the average score of the elderly in their self-assessment of their socio-economic status was 2.3500, indicating that most elderly respondents believed that their socio-economic status was not high, having placed themselves only in the lower-middle class. However, most respondents were “satisfied” with their family income (M = 4.0300), suggesting there is little connection between the elderly’s perception of their socio-economic status and the size of their family income. (3) In terms of the mediating variables, most of the elderly believed that social fairness was at a general level at the time of the survey (M = 3.3841). In terms of the moderating variable, the majority of the elderly believed themselves to be slightly below medium-level social status (M = 4.3667). (4) The results of the control variables show that, from the perspective of individual characteristics, the average age of the interviewees was 70.2349 years old, while the proportion of female interviewees was 48.69%, roughly the same as that of male interviewees. Of the elderly respondents, 77.60% were non-agricultural hukou, and most had a high school education; 32.4% of the interviewees were members of the general public; i.e., party affiliates accounted for the majority of the interviewees. In terms of household characteristics, most interviewees had an average household size of <3 persons and an average annual income of 80,182,000 yuan. Furthermore, major differences were identified between both the household sizes and annual household incomes of the interviewees.

**Table 3:** Definitions of variables and descriptive statistics.

Variables			Description of variables	Mean	SD	Min	Max
Independent variable		Internet use	0 = never; 1 = rarely, sometimes, often, very often	0.7730	0.4190	0	1
Dependent variable	ESG	Economic status	1–5 = lower layer - upper layer	2.3500	0.9031	1	5
		Income satisfaction	1–6 = very dissatisfied - very satisfied	4.0300	0.7654	1	6
	PSG	Political influence	1–4 = strongly disagree–strongly agree	2.0915	0.4196	1	4
	SSG	Food safety	1–6 = very serious–no problem	4.0703	0.8908	1	6
Mediator		SE	1–5 = totally unfair–totally fair	3.3841	0.9622	1	5
Moderator		SS	1–10 = bottom - top	4.3667	1.8643	1	10
Control variables		Gender	0 = female, 1 = male	0.4869	0.4999	0	1
		Age		70.2349	6.878	60	99
		Hukou	0 = farmer, 1 = non-agricultural	0.3240	0.4681	0	1
		Marriage	1 = unmarried, 2 = married, 3 = divorced, 4 = widowed	2.4595	0.9094	1	4
		Education	1 = No education received, 2 = Private schools, literacy classes, 3 = Primary school, 4 = Junior high school, 5 = Vocational high school, 6 = Regular high school, 7 = Vocational school, 8 = Technical school, 9 = College diploma (adult higher education), 10 = College diploma (formal higher education), 11 = Undergraduate degree (adult higher education), 12 = Undergraduate degree (formal higher education), 13 = Graduate student or above	5.7340	1.5942	1	13
		Political affiliation	0 = mass, 1 = partisan	0.1683	0.3742	0	1
		Household size	(Unit: persons)	2.3247	2.1645	0	16
		Income	Annual household income in 2020*0.0001 (10,000 yuan)	8.0182	40.2976	0	999.9908

*p < 0.1.

### 4.2 Model results

#### 4.2.1 Regression analysis

Firstly, to ensure the accuracy of the regression results, the reliability and validity of the measurement questions were analyzed before regression. The results showed that the Cronbach’s alpha value of the perceived variable for the elderly was 0.773, the KMO value of the overall variable was 0.867, and the significance value of the Bartlett sphericity test was 0.000, indicating that the reliability and validity of each variable met the requirements, so the regression analysis could be conducted.

**Figure 2:**
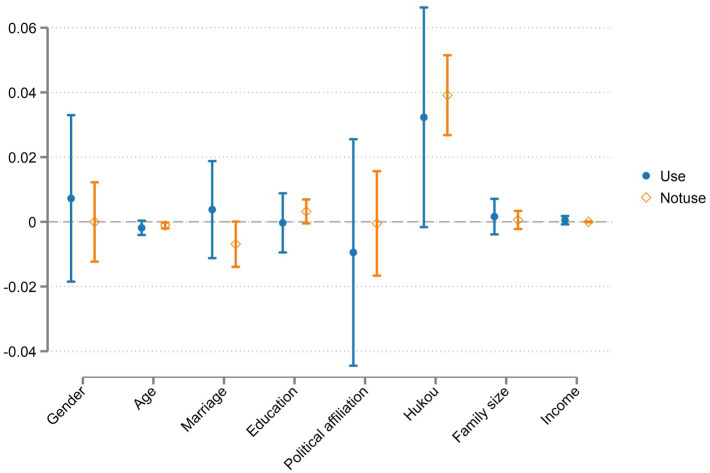
Regression coefficient of whether the elderly use the Internet.

Table 4 reports the basic regression results of this study. In Model 1, after the inclusion of the control variables, Internet use was found to have a significant positive impact on the elderly’s sense of gain, indicating that Internet use can enhance their sense of gain. Assuming that H1a has been established, H1b was unverified. Models 2–4 show the impact of Internet use on the three sub-dimensions of the elderly’s sense of gain (ESG, PSG, and SSG, respectively). According to Model 2, Internet use has a significant positive impact on the sense of economic gain among the elderly, indicating that Internet use can improve the ESG of this group. The results of Model 3 show that Internet use positively affects the elderly’s PSG, but the impact is not significant, which indicates that Internet use fails to improve the PSG of this group. The results of Model 4 show that Internet use has a significant negative impact on SSG. This suggests that the more extensive the use of the Internet, the more likely the SSG is to diminish. In terms of the control variables, multiple individual and household-level variables were also found to have some impact on the sense of access. In terms of the individual-level control variables, the older the person is, the lower their sense of access. Non-agricultural households have a stronger sense of access than agricultural households, and people with party affiliation have a stronger sense of this gain than the masses. As the elderly age, their ability and interest in learning new things gradually decrease (Buchert et al., 2023). Non-agricultural households have greater access to opportunities and resources than agricultural households; those with party affiliation may be affected by identity and other factors, resulting in a stronger sense of access. To more clearly show the differences in the control variables between the elderly who “use the Internet” and those who “do not,” the regression coefficients are plotted in Figure 2.

**Table 4:** Basic regression results.

	Model 1	Model 2	Model 3	Model 4
Internet use	0.0170**	0.0124*	0.0009	−0.0018**
	(0.0090)	(0.0065)	(0.0010)	(0.0009)
Gender	0.0030	0.0022	−0.0012	0.0008
	(0.0053)	(0.0056)	(0.0009)	(0.0007)
Age	−0.0022***	−0.0016***	0.0001	0.0002***
	(0.0006)	(0.0004)	(0.0001)	(0.0001)
Hukou	0.0554***	0.0403***	−0.0002	−0.0013
	(0.0081)	(0.0058)	(0.0009)	(0.0008)
Marriage	−0.0070	−0.0051	0.0001	0.0005
	(0.0044)	(0.0032)	(0.0005)	(0.0004)
Education	0.0033	0.0024	−0.0004	0.0003
	(0.0023)	(0.0017)	(0.0003)	(0.0002)
Political affiliation	0.0023	0.0017	−0.0007	−0.0029***
	(0.0101)	(0.0074)	(0.0012)	(0.0010)
Family size	0.0008	0.0004	−0.0002	0.0007***
	(0.0017)	(0.0012)	(0.0002)	(0.0002)
Income	0.0001	0.0001	−0.0000	0.0000
	(0.0009)	(0.0006)	(0.0000)	(0.0000)
_cons	0.4641***	0.3541***	0.0579***	0.0522***
	(0.0313)	(0.0307)	(0.0049)	(0.0041)
*N*	2,929	2,929	2,929	2,929
Adj. R^2^	0.0023	0.0226	−0.0001	0.0108

*p < 0.1, **p < 0.05, ***p < 0.01, same below.

#### 4.2.2 Mediation model test

To examine whether social equity plays a mediating effect in the process of Internet use affecting the elderly’s sense of access, the stepwise regression analysis described in the previous section was used to conduct the test, with the results shown in Table 5. Model 5 suggests that Internet use has a significant positive effect on the elderly’s sense of gain at the 10% level (Hypothesis H1a was tested further). Model 6 shows that when considering exclusively the sense of social equity, for every unit increase in Internet use, this sense significantly decreases by 0.1365 units (*p* < 0.01). That is, Internet use has a negative inhibitory effect on the elderly’s sense of social equity, so hypothesis H2 was not validated. This may be because the overabundance of information on the Internet means older persons have difficulty in processing it to locate authentic and valuable content. This information overload may not only heighten their cognitive burden but also impair their judgment of social reality, thus adversely affecting their sense of social equity. These issues mean older persons find it difficult to obtain a positive sense of social equity when using the Internet.

**Table 5:** Results of the mediation effect test.

	Model 5	Model 6	Model 7
Internet use	0.0170^**^	−0.1365^***^	0.0156^***^
	(0.0090)	(0.0434)	(0.0065)
Social equity			0.0303^***^
			(0.0028)
Control variables	Yes		
_cons	0.4871^***^	3.9008^***^	0.3459^***^
	(0.0422)	(0.2041)	(0.0326)
*N*	2,929	2,929	2,929
Adj. *R*^2^	0.023	0.007	0.057

**p < 0.05, ***p < 0.01.

Meanwhile, when the mediating variable (sense of social equity) is included in Model 7, the effects of both Internet use and the sense of social equity on the elderly’s sense of gain surpassed the 1% significance level test. Preliminarily, this proves the existence of a mediating effect; i.e., Internet use significantly enhances the elderly’s sense of social equity, which in turn enhances their sense of gain. Thus, hypothesis H3 was preliminarily verified. The robustness of the results was ensured through a Sobel test and the Bootstrap sampling method, the results of which are shown in Table 6. The Sobel test revealed that the Z-statistic of the mediating variable passed the significance test at the 5% level, while the Bootstrap (500) sampling test confidence interval did not contain 0. Based on this analysis, the mediating effect of social justice perception was retained, further verifying hypothesis H3.

**Table 6:** Bootstrap test and Sobel test.

	Coefficient	Std. err.	*t*	P > |t|	95% confidence interval
_bs_1: r (ind eff)	0.0106	0.0054	1.9681	0.0492	[0.0001, 0.0211]
_bs_2: r (dir_eff)	0.0434	0.0039	11.26	0.0000	[0.0358, 0.0510]
Sobel Z	2.0070^**^				

**p < 0.05.

#### 4.2.3 Moderated mediation model test

To test hypothesis H4, the moderating effect of social status on the mediating effect of social equity was analyzed. Referring to Hayes (2013), the analysis was conducted using model 14 in the PROCESS program with 5,000 repetitive extractions, and 95% CI was calculated, as shown in Table 7. The results obtained from the data show that the moderated mediation index has a 95% confidence interval of [−0.0002, 0.0006] and contains 0, indicating that social status did not play a moderating role in social equity affecting access for the elderly; thus, H4 was not supported.

**Table 7:** Moderated mediation model.

Moderator	Conditional indirect effect					Moderated mediation effects test			
	Z	Effect	Boot SE	Boot LLCI	Boot ULCI	Index	Boot SE	Boot LLCI	Boot ULCI
Social status	M-1SD	−0.0013	0.0006	−0.0027	0.0003	0.0002	0.0002	−0.0002	0.0006
	M	−0.0013	0.0005	−0.0021	0.0002				
	M + 1SD	−0.0010	0.0006	−0.0021	0.0005				

### 4.3 Robustness

Given that the analysis above may not have produced robust empirical results due to problems such as an irrational selection of indicators and a biased model setting, various tests were conducted.

#### 4.3.1 Substitution of variable

The measure of the explanatory variable of Internet use was replaced with questionnaire question A30b: “In the last 6 months, have you gone online, including using various devices such as computers, cell phones, and smart wearables to go online.” The results are shown in column (1) of Table 8. Although the regression results of columns (2–4) are slightly different from those shown in Table 2, the facilitating effect of Internet use on the elderly’s sense of gain was still robust (*p* < 0.01), and the estimation results were consistent with those of the previous paper, indicating that the baseline regression results are robust.

**Table 8:** Robustness tests: replacing explanatory variables.

	(1)	(2)	(3)	(4)
Past use	0.0152***	0.0142**	0.0007	0.0003
	(0.0058)	(0.0057)	(0.0009)	(0.0008)
Sex	0.0025	0.0029	−0.0012	0.0008
	(0.0057)	(0.0056)	(0.0009)	(0.0007)
Age	−0.0011***	−0.0014***	0.0001	0.0002***
	(0.0004)	(0.0004)	(0.0001)	(0.0001)
Marriage	−0.0040	−0.0045	0.0001	0.0004
	(0.0032)	(0.0032)	(0.0005)	(0.0004)
Education	0.0026	0.0027	−0.0003	0.0002
	(0.0017)	(0.0017)	(0.0003)	(0.0002)
Political	−0.0023	0.0014	−0.0008	−0.0029***
	(0.0075)	(0.0073)	(0.0012)	(0.0010)
Hukou	0.0398***	0.0414***	−0.0001	−0.0015*
	(0.0059)	(0.0058)	(0.0009)	(0.0008)
Family size	0.0009	0.0005	−0.0002	0.0007***
	(0.0013)	(0.0012)	(0.0002)	(0.0002)
Income	0.0000	0.0000	−0.0000	0.0000
	(0.0001)	(0.0001)	(0.0000)	(0.0000)
_cons	0.4482***	0.3396***	0.0573***	0.0513***
	(0.0321)	(0.0314)	(0.0050)	(0.0042)
*N*	2,929	2,929	2,929	2,929
Adj_*R*^2^	0.023	0.006	0.060	0.0003

*p < 0.1, **p < 0.05, ***p < 0.01.

#### 4.3.2 Substitution of model

The Ologit model was used to replace the OLS model for robustness testing. Since the explanatory variables in this study are ordered variables, the Ologit model was used for regression analysis. In Table 9, Models 1 and 2 are the regression analysis results of the original and Ologit models, respectively. The coefficients of the explanatory variables were found to be positive, and the effects on the sense of access among the elderly were all significant at the 5% level, so the regression results are highly consistent. Meanwhile, the coefficients of the mediating variables are positive and negative, and the degree of significance is also consistent with the regression results of the original model, verifying that the aforementioned model has good robustness.

**Table 9:** Robustness tests: replacing model.

	Model 1 OLS	Model 2 Ologit
Internet use	0.0170** (0.0090)	0.1467** (0.0683)
Social equity	0.0303^***^ (0.0028)	0.3506*** (0.0347)
Control variables	Yes	
*N*	2,929	

**p < 0.05, ***p < 0.01.

### 4.4 Heterogeneity analysis

#### 4.4.1 Household income heterogeneity

Household income is one of the most important factors influencing older people’s Internet use behavior and sense of gain (Zhao et al., 2024). Therefore, to examine the heterogeneity of the impact of Internet use on the elderly’s sense of gain across household income levels, the household incomes of this segment were categorised into three quartiles: low, medium, and high (Xie et al., 2024). The results are shown in columns (1–3) of Table 10. In terms of household income heterogeneity, the regression results in these columns show that Internet use has no significant effect on the sense of gain among elderly people with middle and high levels of income, while it has a significant positive effect on this dimension among elderly people with low incomes, at the 1% level. This suggests that Internet use is conducive to enhancing the sense of gain among the low-income group. One reason for this could be that poorer older adults are more likely than their middle- and high-income counterparts to encounter information isolation and limited access to resources (Luiu and Tight, 2021). The Internet provides the poorer group with a platform to socialize, interact, and access information and perhaps even telework opportunities (Rony et al., 2024). These activities can be crucial in enhancing their quality of life. Access to these resources is vital in improving the quality of life of low-income elderly people, which can subsequently enhance their sense of gain.

**Table 10:** Heterogeneity in household income and education level.

	Income: (1–3)			Education: (4–6)		
	(1) Low-income	(2) Middle-income	(3) High-income	(4) Low literacy	(5) Middle literacy	(6) High literacy
Internet use	0.0335***	0.0093	−0.0196	−0.0067	0.0029	0.0144*
	(0.0117)	(0.0097)	(0.0146)	(0.0204)	(0.0247)	(0.0074)
Control variables	Yes					
_cons	0.5336***	0.4330***	0.4698***	0.3449***	0.4754***	0.5203***
	(0.0584)	(0.0447)	(0.0675)	(0.0665)	(0.0768)	(0.0390)
*N*	977	1,365	587	269	224	2,436
Adj. *R*^2^	0.0278	0.0203	0.0403	0.0079	0.0114	0.0230

*p < 0.1, ***p < 0.01.

#### 4.4.2 Heterogeneity in education levels

For older adults, their education level is a key determinant of their ability to use the Internet (Jung et al., 2022; Shi et al., 2023). In general, attaining a higher level of education makes elderly people more likely to acquire Internet skills and be able to utilize this platform more effectively to meet their needs. With reference to previous studies (Zhao and Zhang, 2025), the education levels of older adults were arranged into three categories, with the results presented in columns (4–6) of Table 10. The analysis demonstrated that Internet use positively and significantly affects the sense of gain among the elderly belonging to the high literacy group at the 10% level. Meanwhile, this group are better able to use the Internet, thus effectively improving their sense of gain. One reason for this could be that highly literate elderly people usually have strong learning and information-processing abilities (Lei et al., 2024). These enable their rapid adaptation to and mastery of the new technology of the Internet before they utilize the services and resources it provides, thus enhancing their sense of gain.

#### 4.4.3 Urban–rural hukou heterogeneity

Differences between urban and rural areas regarding economic development and resource allocation means older adults may differ significantly in terms of Internet access and use, depending on the type of region they live in. In view of this, group regressions were conducted using household registrations to examine how Internet use impacts the sense of gain among elderly people holding different types of registration. As columns (7, 8) of Table 11 illustrate, Internet use can positively and significantly affect the sense of gain among the urban elderly at the 10% level. This suggests that among urban elderly, the sense of gain is significantly enhanced as their Internet use deepens. This may be because they generally live in areas with better infrastructures, so they use the Internet more (Ma, 2024). Thus, they are more likely to utilize the resources it offers, such as online education and telemedicine, to enhance their quality of life and sense of gain.

**Table 11:** Heterogeneity in urban and rural households and marital status.

	Urban–rural		Spouse	
	(7) Rural hukou	(8) Urban hukou	(9) Widowed/divorced	(10) With spouse
Internet use	0.0071	0.0160*	−0.0083	0.0184**
	(0.0100)	(0.0093)	(0.0128)	(0.0078)
Control variables	Yes			
_cons	0.3736***	0.3728***	0.4034***	0.4884***
	(0.0198)	(0.0218)	(0.0587)	(0.0385)
*N*	1,711	1,218	847	2,082
Adj. *R*^2^	0.0205	0.0095	0.0131	0.0231

*p < 0.1, **p < 0.05, ***p < 0.01.

#### 4.4.4 Marital status heterogeneity

Among older adults with spouses, Internet use is often shared between couples for activities such as online entertainment or information access. These shared experiences can enhance their emotional connection and sense of gain (Marler and Hargittai, 2024; Bröning and Wartberg, 2022). Therefore, marital status was employed in this study to test the heterogeneity of the elderly’s sense of gain. As shown in columns (9, 10) of Table 11, older adults with spouses can enhance their sense of gain more significantly when using the Internet compared to divorced and widowed elderly people This may be because elderly couples can share information and discuss topics during their joint Internet use. This interaction promotes their emotional communication, helping to alleviate their sense of loneliness and enhancing their sense of gain. In contrast, divorced and widowed older people may be at higher mental health risks, such as being more likely to feel lonely (Wang and Shi, 2024). This psychological state may affect the Internet use experiences of this group and perhaps even reduce their Internet use, which would further limit the impact of Internet use on their sense of acquisition.

## 5 Discussion

On the basis of the two-dimensional differentiation of Internet use (using the Internet and not using the Internet), this study discusses the influence of Internet use on the sense of gain among the elderly and its mechanism from the logic of Internet technology empowerment, while a theoretical framework was also constructed. Using the data from CGSS2021, the indicators were comprehensively evaluated using the entropy method, while the OLS model, mediating effect, and moderating effect models were later applied to test the relationship between the perceptions of social equity and social status on the Internet and the sense of accessibility among the elderly. The study revealed various key findings.

Firstly, Internet use was found to have a significant positive effect on the elderly’s sense of gain; that is, elderly Internet users might have a higher sense of gain, so H1a was confirmed. This result provides empirical support for the capacity of Internet technology to cope with an aging society, and it corroborates the findings of related works. Heo et al. (2015) found that Internet use can generate positive emotions in the elderly, such as contentment and happiness. More specifically, the digital dividend created by the development of the Internet can expand the economic structure and material affluence of the elderly (He et al., 2022; Li, 2019), while the elderly enjoy more benefits through the use of specific Internet functions, thus increasing their economic sense of gain. In the political sense of gain, the influence of Internet use is not significant for two possible reasons. First, the digital divide is more obvious in Internet use among the elderly (Leite et al., 2024). For example, elderly people may be unable to use the Internet to participate smoothly in political activities due to internal and external factors such as physiological degradation and the complexity of Internet operation, which prevents this group from using the Internet to enhance their political sense of gain. On the other hand, an individual’s political perception might be related to political knowledge linkage but not Internet use (Loy et al., 2019), so Internet use does not significantly enhance the political sense of gain among the elderly. In terms of security access, although the Internet provides a convenient way for the elderly to participate in politics (Vicente and Novo, 2014), online privacy and cybersecurity issues are of greater concern among cautious older adults (Bandalović et al., 2022; Liu, 2022).

Secondly, Internet use has a significant negative impact on the perception of social equity among the elderly, this is inconsistent with hypothesis 2, therefore hypothesis 2 loses validation. A possible reason for this is the major differences in the screening, understanding, and use of information by older people with different characteristics when using the Internet. Such differences are more likely to be amplified in the Internet context, resulting in social equity among the elderly deviating from reality. This finding can be corroborated by the related research. Zhu et al. (2020) argued that Internet technology increases social transparency, which intentionally or unintentionally expands the scope of social comparison among the elderly, thus negatively affecting their social equity. Li (2019) found that the negative effect of the social equity brought about by Internet use deserves greater attention. In addition, the digital divide is one of the direct causes of perceptions of social inequity.

Thirdly, a sense of social equity can play a mediating role in the impact of Internet use on older people’s sense of access. Compared with traditional media, the Internet is characterized by fast dissemination and large amounts of information, which can make older people pay more attention to social equity issues. When the elderly feel they experience social fairness, they are more likely to have a positive mindset and positively evaluate their living conditions, thus increasing their sense of acquisition. Conversely, if this group feels they experience social injustice, such negative feelings may affect their overall evaluation of their own lives, resulting in their developing a low sense of gain. This conclusion is similar to the findings obtained by Yang et al. (2023) and Ren et al. (2022).

Finally, social status does not significantly moderate the relationship between Internet use and a sense of social equity. This conclusion is partially consistent with the results obtained by Kang et al. (2024). One possible reason is that, on the one hand, as a platform for information exchange and dissemination, actual Internet application use by the elderly may also affect the moderating effect of social status on the sense of gain. For example, the elderly may pay more attention to the acquisition of information and the maintenance of social interaction when using the Internet, rather than the promotion or confirmation of social status, which may weaken the regulating effect of social status on the sense of gain. On the other hand, the sense of gain among the elderly may also be affected by other factors such as health status, family relationships, and social support. These might influence more significantly the process of Internet use affecting the sense of gain among the elderly through the sense of social justice, thus concealing the moderating effect of social status, resulting in a less significant moderating effect. In addition, the heterogeneity test revealed that access was more likely to be positively affected by Internet use among four groups of older adults: those with low household income levels, higher levels of literacy, urban hukou, and spouses.

This research reveals the relationship between Internet use and the sense of elderly gain to a certain extent, but various limitations remain. First, cross-sectional data was used, which has the drawback of making it difficult to identify exactly the causal effect. Therefore, panel data could be used in future studies to clarify the relationship between the variables. Second, the measurement of the explanatory variables (sense of gain), mediating variables, and moderating variables in this research derived from a subjective evaluation of the interviewees, so bias due to subjectivity might have occurred. This may have affected the accuracy of the regression results, so a follow-up study could further analyze these from other levels.

## 6 Conclusion

In this study, empirical analysis was conducted to determine the impact of Internet use on the elderly’s sense of gain using the OLS model. Furthermore, the mediating and moderating effects of social equity and social status were explored. Several main conclusions were drawn.

Firstly, Internet use can significantly promote the elderly’s sense of gain. Internet use was demonstrated to positively and significantly enhance the sense of economic gain; while it positively affects the sense of political gain, the effect is not significant. Internet use has a significant negative effect on the sense of security gain. In this regard, the relevant government departments are recommended to guide the elderly on effectively utilizing the Internet to enhance their economic gains; to develop more elderly-friendly government service platforms; and to increase this group’s participation in and understanding of political activities.

Second, the sense of social equity plays a mediating role between Internet use and the elderly’s sense of gain. Older adults receive a wide variety of information through the Internet and then make judgments about the degree of social equity, which ultimately affects their sense of gain. The regression results remained robust after the measurement problem of the explanatory variables was substituted with the replacement model. The government should regulate online information more stringently to prevent misleading information from potentially harming the social equity of the elderly.

Third, in different sample subgroups, Internet use had heterogeneous effects on the elderly’s sense of gain when these people were categorized by household income, education level, urban and rural hukou, and marital status. More specifically, Internet use had greater impacts on the sense of gain among older people who had low household incomes, high education levels, urban hukou, and/or spouses. To address this, the government and relevant departments should invest more in rural Internet infrastructure to help eliminate the urban–rural digital divide. Meanwhile, the children of divorced older persons without should give their parents more care and companionship to reduce their sense of isolation.

## Data Availability

The original contributions presented in the study are included in the article/supplementary material, further inquiries can be directed to the corresponding author.
